# The PI3K/AKT Pathway and FOXO3a Transcription Factor Mediate High Glucose-Induced Apoptosis in Neonatal Rat Ventricular Myocytes

**DOI:** 10.5812/ircmj.14914

**Published:** 2014-04-05

**Authors:** Weiguo Bao, Feng Pan, Ling Chen, Guohai Su, Xiaoyuan Gao, Ying Li, Qiang Sun, Jinhui Sun, Kun He, Hui Song

**Affiliations:** 1 Department of Cardiac Surgery, Second Hospital of Shandong University, Jinan, PR China; 2 Department of Cardiology, Jinan Central Hospital of Shandong University, Jinan, PR China; 3 School of Public Health, Shandong University, Jinan, PR China

**Keywords:** Myocytes, Cardiac, Apoptosis, gleditsioside B

## Abstract

**Background:**

PI3K/AKT pathway plays major roles in regulating cardiomyocyte metabolism. The roles of PI3K/AKT pathway and FOXO3a in mediating high glucose-induced apoptosis in cardiomyocytes remain unclear.

**Objectives:**

In this experimental study, we investigated the mechanisms of the PI3K/AKT pathway and FOXO3a in mediating hyperglycemia-induced apoptosis in neonatal rat ventricular myocytes (NRVMs).

**Materials and Methods:**

NRVMs were adopted as the cell model to investigate the roles of PI3K/AKT and FOXO3a in mediating hyperglycemia-induced apoptosis in cardiomyocytes. Annexin-V-FITC staining and PI staining were used to evaluate the apoptosis in NRVMs under indicated conditions of serum starvation, high glucose exposure, and pharmacological or genetic manipulations on the expressions of PI3K/AKT and FOXO3a. Western blotting was conducted to evaluate the cytoplasmic/nuclear localization of FOXO3a in NRVMs exposed to high glucose. FOXO3a transcriptional activity was measured by luciferase reporter assay.

**Results:**

High glucose (30 mM) induced significant apoptosis in serum-starved NRVMs as compared with normal glucose (5 mM) control (12.01 ± 0.76% vs. 2.86 ± 0.55%; P < 0.001). Treatment with IGF1 attenuated hyperglycemia-induced apoptosis by 68% (3.23 ± 0.76% vs. 9.97 ± 1.29%; P < 0.001; n = 3) in comparison with the non-treated control. Treatment with PI3K inhibitor LY294002 enhanced hyperglycemia-induced apoptosis by 109% (20.83 ± 1.87% vs. 9.97 ± 1.29%; P < 0.001; n = 3) in comparison with the non-treated control. Over-expression of AKT by transduction with CA-AKT attenuated hyperglycemia-induced apoptosis by 47% (5.48 ± 0.35% vs.10.31 ± 0.94%; P < 0.001; n = 3) in comparison with the empty-vector control. Transduction with DN-AKT enhanced high glucose-induced apoptosis by 105% (21.13 ± 1.11% vs. 10.31 ± 0.94%; P < 0.001; n = 3) in comparison with the empty-vector control. Western blotting showed that high glucose induced a significant increase in FOXO3a nuclear localization. Luciferase reporter assay showed that high glucose induced a significant increase of 310% (P < 0.001; n = 3) in FOXO3a transcriptional activity against Fas ligand when NRVMs were transducted with TM-FOXO3a in comparison with the empty-vector control.

**Conclusions:**

The PI3K/AKT pathway mediated hyperglycemia-induced apoptosis of NRVMs through the translocation of FOXO3a to nuclei and the resultant enhanced transcriptional activity of FOXO3.

## 1. Background

Hyperglycemia is considered a major pathogenic factor causing abnormalities at the cardiac myocyte level, eventually leading to structural and functional abnormalities (1). Oxidative stress has been shown to play a role in pathogenesis and progression of cardiomyocyte apoptosis (2). JNK signaling pathway is activated by hyperglycemia-induced oxidative stress (3). Other research suggested that high glucose induces apoptosis in cardiomyocytes through activation of caspase-3 (4). The Forkhead class O (FOXO) transcription factors are downstream effectors of AKT, consisting of four subfamily members, including FOXO1 (FKHR), FOXO3a (FKHRL1), FOXO4 (AFX) and FOXO6 (5). FOXO transcriptional factors are involved in diverse activities, including response to oxidative stress (6), regulation of metabolism (7) and apoptosis (8). FOXO transcriptional factors, together with PI3K/AKT signaling have been shown as important determinants in the homeostasis of cardiac myocytes (9). However, the mechanism underlying hyperglycemia-induced apoptosis of cardiomyocytes remains unclear.

## 2. Objectives

We aimed to identify the mechanism by which PI3K/AKT pathway and FOXO3a mediate high glucose-induced apoptosis. Therefore, we performed the study by examining four following questions sequentially:

1. the extent to which high glucose induces apoptosis;
2. whether up-regulating or down-regulating PI3K/AKT pathway affects glucose-induced apoptosis;
3. whether the sub-cellular localization and expression of FOXO3a are affected by high glucose exposure; and
4. whether high glucose exposure causes enhanced FOXO3a transcriptional activity.

## 3. Materials and Methods

### 3.1. Neonatal Cardiomyocyte Isolation

In this experimental study, the procedures and protocols involving animals were approved by the Animal Use Committee of Shandong University. Neonatal rat ventricular myocytes (NRVMs) were isolated as previously published (2) with slight modifications. Briefly, pregnant Wistar rats were kept in an air-conditioned room at 21°C with a relative humidity of 55% and a 12-hour light cycle. The pregnant rats were fed with standard rodent chow, and water was given ad libitum until delivery. Two days after birth, six neonatal rats were killed, and NRVMs were isolated from the neonatal rats using a commercial neonatal cardiomyocyte isolation system (Worthington Biochemical Corporation, USA) according to the manufacturer’s instructions. The cells were then preplated after random allocation for two hours for further treatment in Dulbecco's modified Eagle medium (DMEM, GIBCO) supplemented with 10% fetal bovine serum (FBS, GIBCO), containing 1% antibiotics (penicillin and streptomycin), and then plated in a humid atmosphere of 5% CO2 plus 95% air.

### 3.2. Plasmid Constructs and Adenovirus Preparation

Adenoviral vectors expressing wild type AKT (WT-AKT), dominant negative AKT (DN-AKT) and constitutively active AKT (CA-AKT), which were tagged with the HA-epitope, were constructed as described previously (5). The DN-AKT has alanine residues substituted for threonine at position 308 (Thr308) and serine at position 473 (Ser473). The CA-AKT has the c-Src myristoylation sequence fused in-frame to the N terminus of the WT-AKT coding sequence, which targets the fusion protein to the membrane. Adenoviral vectors encoding wild-type FOXO3a (WT-FOXO3a) and a nonphosphorylatable, constitutively active form of FOXO3a (TM-FOXO3a) were described previously (10). All constructs were amplified in 293 cells and purified by ultracentrifugation with viral titers determined as plaque-forming units (9). For adenoviral transduction, cardiomyocyte cultures were incubated with adenovirus at a multiplicity of infection of 10-50 for 12 hours.

### 3.3. Cell Culture and Treatment

NRVMs were cultured and treated as previously reported with slight modifications (2). In brief, NRVMs were grown in modified DMEM (10% FBS, 1% penicillin, and 1% streptomycin) supplemented with 5 mM glucose (Sigma) for 24 hours following isolation. For apoptosis assay, the cells were then incubated in fresh media of either the modified DMEM or serum-free DMEM treated with 5 mM glucose, 15 mM glucose, or 30 mM glucose in the presence or absence of pretreatment with growth factor IGF1 (50 ng/mL, Sigma). In some experiments, NRVMs were pretreated with adenoviral transfection to overexpress AKT expression or pretreated with PI3K inhibitor LY294002 (50 nM, Sigma) or Wortmannin (100 nM, Sigma) before high glucose treatment. The osmolality of all culture media were equal to 30 mM by adding different amounts of mannitol (Sigma), and all culture media contained 1% penicillin and streptomycin (Sigma).

### 3.4. Apoptosis Assay

The NRVMs were harvested after indicated treatments with trypsin (0.25%) and a single cell suspension prepared. Cells were then washed with PBS and pelleted by centrifugation at 1200 rpm for 5 minutes. Cells were resuspended in binding buffer and the cell density was adjusted to 5 × 10^5^ cells ⁄ ml. A 95-µL aliquot of the cell suspension was added to 5 µL Annexin-V-FITC, and then cells were incubated for 10 minutes at room temperature in the dark. The suspension was then washed with PBS and resuspended in 190 µL binding buffer before adding 10 µL propidium iodide (PI) to obtain a final concentration of 1 µg ⁄mL PI. The samples were examined by flow cytometry (BD FACSVantage; BD Sciences, San Jose, CA, USA). The results were analyzed using cell quest software (BD Sciences) to determine the rate of apoptosis in the lower right quadrant.

### 3.5. Sub-cellular Fractionation and Western Blotting

For nuclear/cytoplasmic fractionation, cultured NRVMs were fractionated into nuclear and cytoplasmic lysates using a PARIS kit (Ambion) according to manufacturer’s instructions. Western blots were performed as described previously (9) with slight modifications. Briefly, tissue samples were homogenized in lysis buffer. A total of 20-50 µg of proteins was transferred to a PVDF (BioRad) membrane through 12% SDS-PAGE. The primary antibodies used in this study included rabbit anti-FOXO3a polyclonal antibody (1:500, Santa Cruz), anti-phospho-FOXO3a polyclonal antibody (Ser253, 1:500, Santa Cruz), and GAPDH (1:2000, Cell Signaling). Signals of bound antibodies were developed by enhanced chemiluminescence (Amersham). NIH ImageJ software was used to measure the densities of protein signals on X-ray films. The immunoblot intensity was normalized to the loading control GAPDH.

### 3.6. Luciferase Reporter Assay

The FOXO3a expression vectors (wild type and triple mutants) and FOXO luciferase constructs were described previously (11). NRVMs were transfected for 12 hours with empty vector, or triple mutants FOXO3a (TM-FOXO3A) along with reporter plasmids (FHRE-Luc) under the control of a fragment of the Fas ligand promoter containing the FOXO3a binding site as previously reported (5). The transfection medium was then replaced with serum free DMEM and then NRVMs were treated with either normal glucose or high glucose. After incubation for 24 hours, Luciferase activity was determined using a Luciferase assay kit (Promega) and was normalized for transfection efficiency using a β-galactosidase-expressing vector.

### 3.7. Statistical Analysis

The statistical analyses were performed using SAS software (version 9.1 under Windows XP). Data were analyzed by General Linear Model using treatments and glucose doses as class factors. Student’s t-test was performed when appropriate. Results were represented by mean ± SD. Assumptions of normality and homoscedasticity were checked. P < 0.05 was considered significant.

## 4. Results

### 4.1. High glucose Induced Apoptosis in NRVMs in a Dose and Serum-Dependent Manner

To investigate whether glucose concentration and serum affect apoptosis in NRVMs, the cells were treated with glucose at three different concentrations (5, 15, and 30 mM) in DMEM supplemented or not with 10% FBS for 24 hours. The apoptosis was then assessed by Annexin-V-FITC (Table 1). When NRVMs were incubated in DMEM plus 10% FBS, three concentrations of glucose induced similar apoptosis indices (F = 0.17; P = 0.844). When NRVMs were incubated in serum-free DMEM, the concentration of glucose showed significant effects (F = 237.88; P < 0.001). The apoptotic index in serum-starved NRVMs treated with normal glucose (5 mM) showed an increase of 140% (2.86 ± 0.55% vs. 1.19 ± 0.15%; P = 0.003) compared with the serum-stimulated NRVMs treated with normal glucose. The apoptotic index in serum-starved NRVMs treated with high glucose (30 mM) represented a marked increase of 734% (12.01 ± 0.76% vs. 1.44 ± 0.24%; P < 0.001) as compared with serum-stimulated NRVMs treated with high glucose. Taken together, high glucose induced apoptosis in NRVMs in a dose and serum dependent manner. Thereafter, the apoptotic indices of NRVMs exposed to 5 mM and 30 mM glucose under serum starvation for 24 hours were referred to as respective basal apoptotic indices.

**Table 1.** High Glucose Induced Apoptosis in NRVMs in a Dose and Serum-Dependent Manner (n = 3) ^a^

FBS, %	Apoptotic Index at 24 hours, %				
	Glucose Dose, mM			F	P Value of ANOVA
	5	15	30		
**10**	1.19 ± 0.15	1.25 ± 0.25	1.44 ± 0.24	0.17	0.844
**0**	2.86 ± 0.55 ^b^	7.20 ± 0.90 ^b,c^	12.01 ± 0.76 ^b^, ^c^	237.88	< 0.001

^a^ Data are presented in Mean ± SD.

^b^ P < 0.05 or P < 0.01 compared with the group in the same column.

^c^ P < 0.05 or P < 0.01 compared with normal glucose control in the same row.

### 4.2. PI3K/AKT Pathway Mediated High Glucose-Induced Apoptosis in NRVMs

Since PI3K/AKT pathway was mediated by the growth factor IGF1 in NRVMs (12), we next examined the effects of pharmacological manipulations on PI3K/AKT pathway in mediating apoptosis. NRVMs were incubated for 2 hours in serum-free DMEM treated or not with IGF1, specific PI3K inhibitor (LY294002), or Wortmannin and then incubated with either normal glucose (5 mM) or high glucose (30 mM) for 24 hours. Apoptosis was then assessed by Annexin-V-FITC (Table 2). Treatment with IGF1 attenuated apoptosis significantly by 72% (0.97 ± 0.17% vs. 3.50 ± 0.22%; P < 0.019) or 68% (3.23 ± 0.76% vs. 9.97 ± 1.29%; P < 0.001) in NRVMs exposed to either normal or high glucose compared with the non-treated controls. In contrast, inhibition of PI3K/AKT pathway by LY294002 enhanced apoptosis by 115% (7.53 ± 0.34% vs. 3.50 ± 0.22%; P < 0.001) or 109% (20.83 ± 1.87% vs. 9.97 ± 1.29%; P < 0.001) in NRVMs exposed to either normal or high glucose as compared with the non-treated controls. Wortmannin showed similar effects as LY294002. Collectively, pharmacological manipulations on PI3K/AKT pathway mediate hyperglycemia-induced apoptosis in NRVMs. In parallel, we also investigated genetic manipulations on the PI3K/AKT pathway in hyperglycemia-induced apoptosis by mediating AKT expression using adenoviral transduction with wild type AKT (WT-AKT), constitutively active AKT (CA-AKT), or dominant negative AKT (DN-AKT). NRVMs were transfected for 12 hours with empty vector, or plasmid expressing WT-AKT, CA-AKT, or DN-AKT and then incubated in serum-free DMEM supplemented with either normal glucose (5 mM) or high glucose (30 mM) for 24 hours. The apoptosis was then evaluated by Annexin-V-FITC (Table 3). Transduction with empty vector had no significant effects on apoptosis in NRVMS exposed to either normal (3.53 ± 0.38% vs. 2.86 ± 0.55%; P = 0.092) or high (10.31 ± 0.94% vs. 12.01 ± 0.76%; P = 0.088) glucose compared with the respective basal apoptotic indices.

Overexpression of AKT by transduction with WT-AKT or CA-AKT significantly inhibited apoptosis by 61% (1.37 ± 0.46% vs. 3.53 ± 0.38%; P = 0.008) or 63% (1.29 ± 0.44% vs. 3.53 ± 0.38%; P = 0.006) in NRVMs exposed to normal glucose (5 mM) in comparison with the empty vector controls. Overexpression of AKT by transduction with WT-AKT or CA-AKT significantly inhibited apoptosis by 30% (7.20 ± 0.97% vs. 10.31 ± 0.94%; P = 0.001) or 47% (5.48 ± 0.35% vs.10.31 ± 0.94%; P < 0.001) in NRVMs exposed to high glucose (30 mM) in comparison with the empty vector controls. In contrast, DN-AKT significantly enhanced apoptosis by 193% (10.36 ± 1.52% vs. 3.53 ± 0.38%; P < 0.001) or 105% (21.13 ± 1.11% vs. 10.31 ± 0.94%; P < 0.001) in NRVMs exposed to either normal or high glucose compared with the empty vector controls. Taken together, the PI3K/AKT pathway mediated hyperglycemia-induced apoptosis in NRVMs and the mediating effects were subject to regulations by both pharmacological and genetic means manipulating the PI3K/AKT pathway.

**Table 2.** Pharmacological Manipulations on the PI3K/AKT Pathway Mediated High Glucose Induced Apoptosis in NRVMs (n = 3) ^a^

Glucose, mM	Apoptosis Index, %					
	Treatment					
	Control	IGF1	LY294002	Wortmannin	F	P Value of ANOVA
**5**	3.50 ± 0.22	0.97 ± 0.17 ^b^	7.53 ± 0.34 ^c^	7.59 ± 0.98 ^c^	22.51	< 0.001
**30**	9.97 ± 1.29 ^d^	3.23 ± 0.76 ^c^, ^e^	20.83 ± 1.87 ^c^, ^d^	21.52 ± 2.08 ^c^, ^d^	167.95	< 0.001

^a^ Data are presented in Mean ± SD.

^b^ P < 0.05 compared with the non-treated control in the same row.

^c^ P < 0.01 compared with the non-treated control in the same row.

^d^ P < 0.01 compared with the group in the same column.

^e^P < 0.05 compared with the group in the same column.

**Table 3.** Genetic Manipulations on the PI3K/AKT Pathway Mediated High Glucose Induced Apoptosis in NRVMs (n = 3) ^a^

Glucose, mM	Apoptosis Index, %					
	Treatment					
	EMP	WT-AKT	CA-AKT	DN-AKT	F	P Value of ANOVA
**5**	3.53 ± 0.38	1.37 ± 0.46 ^b^	1.29 ± 0.44 ^b^	10.36 ± 1.52 ^b^	72.74	< 0.001
**30**	10.31 ± 0.94 ^c^	7.20 ± 0.97 ^b^, ^c^	5.48 ± 0.35 ^b^, ^c^	21.13 ± 1.11 ^b^, ^c^	196.35	< 0.001

^a^ Data are presented in Mean ± SD.

^b^ P < 0.05 and P < 0.01 compared with the empty-vector control in the same row.

^c^ P < 0.05 and P < 0.01 compared with the group in the same column; EMP: the empty-vector control.

### 4.3. High Glucose Exposure Resulted in Translocation of FOXO3a to Nuclei

**Figure 1.**
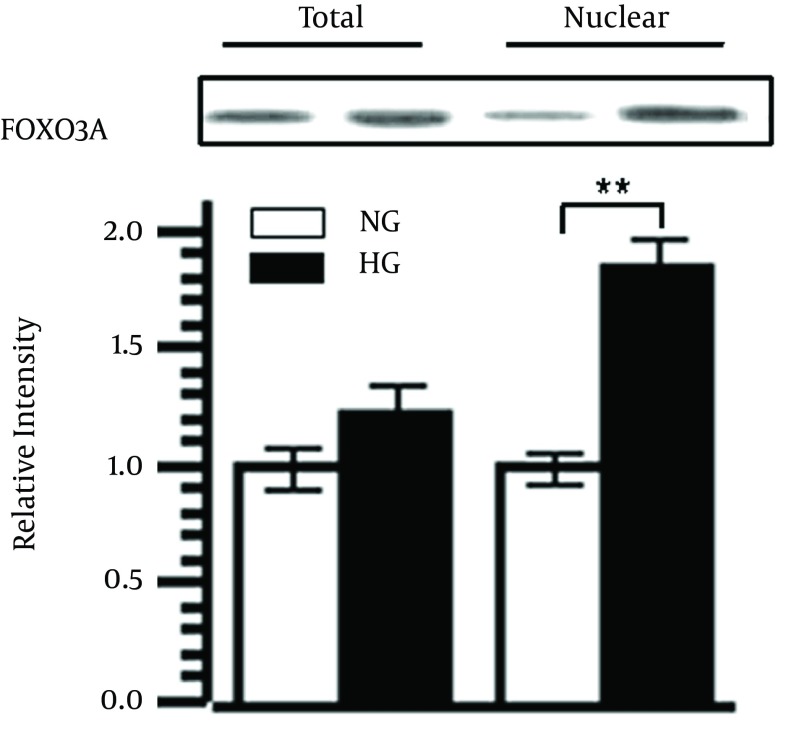
High Glucose Exposure Resulted in Translocation of FOXO3a to Nuclei NRVMs were incubated in serum free DMEM treated with either normal glucose (5 mM) or high glucose (30 mM) for 24 hours. The expression level of total FOXO3a as well as nuclear FOXO3a was examined by subcellular fractionation followed by Western blotting and then the densitometry was analyzed quantitatively. The expression of total FOXO3a and nuclear FOXO3a were normalized to loading control GAPDH. Data represent mean ± SD (n = 3). ** P < 0.01 versus respective normal glucose controls.

There is evidence that the localization of FOXO3a to nuclei activates genes associated with cell proliferation, metabolism, and apoptosis (9). Therefore, we next examined the effects of high glucose on the localization of cellular FOXO3a by measuring the total cellular FOXO3a and nuclear FOXO3a. Serum-starved NRVMs were treated with either normal (5 mM) or high (30 mM) glucose for 24 h. The expression of the total FOXO3a and nuclear FOXO3a were measured using subcellular fractionation followed by Western blotting as mentioned above. The results were quantified by densitometric analysis (Figure 1). High glucose induced a slight increase of total FOXO3a compared with the normal glucose control (P = 0.159). In contrast, high glucose induced a significant increase (169% of control; P = 0.005) of FOXO3a nuclear localization as measured at 24 hours compared with the normal glucose control. These data suggested that high glucose exerted its effects on apoptosis through translocation of FOXO3a from cytoplasm to nuclei.

### 4.4. Inhibition of PI3K/AKT Pathway and Phosphorylation Deficient Mutants of FOXO3a Enhanced FOXO3a Transcriptional Activity

We next examined whether high glucose exposure enhances hyperglycemia-induced FOXO3a transcriptional activity. Serum-starved NRVMs were first transfected with empty vector, or plasmid expressing triple mutants FOXO3a (TM-FOXO3a) for 12 hours, followed by high glucose treatment (30 mM) for an additional 24-hour. FOXO3a transcriptional activity was then measured by Lucifer reporter assay. High glucose induced significant increase (410% of control; P < 0.001; n = 3) in FOXO3a transcriptional activity against Fas ligand when NRVMs were transfected with TM-FOXO3a compared with empty vector control (data not shown), which confirmed that FOXO3a played roles in hyperglycemia-induced apoptosis in NRVMs.

## 5. Discussion

Since AKT bears close relationships to a variety of effectors relating to apoptosis (13), it is reasonable to hypothesize that AKT plays roles in cardiomyocyte apoptosis caused by high glucose exposure. NRVM was employed as the cell model to investigate hyperglycemia-induced apoptosis, since this cell line is well defined with respect to the PI3K/AKT pathway as well as the expressions of FOXO transcription factors (5). Yet the results in this study cannot be applied directly to adult rat myocytes, considering the differential expressions of FOXO factors in neonatal and adult rat myocytes (5).

The extent to which high glucose induces apoptosis in NRVMs under serum stimulation or serum starvation for 24 hours was evaluated. Meanwhile, the apoptosis of NRVMs exposed to high glucose with or without pharmacological or genetic manipulations on PI3K/AKT expression, which is an established method to investigate the effects of PI3K/AKT pathway on its presumable down-stream effectors (5, 14, 15). Furthermore, these two sets of experiments measuring apoptosis under indicated conditions were conducted in parallel, since the FOXO transcription factors decline gradually during the neonatal stage and disappear at neonatal 7th day (14). Therefore, the incubation time of 24 hours is more appropriate as compared with the incubation time of 48 hours. A recent breakthrough revealed that FOXO3a exerts feedback control over upstream signaling including the PI3K/AKT pathway, which was observed in cardiomyocytes exposed to normal glucose (15). Nevertheless, how this feedback control interacts with high glucose exposure in neonatal or adult cardiomyocytes remains unclear. Furthermore, it has been reported that there are two phosphorylation sites for AKT at either Thr308 or Ser473, which behave differentially under physiological and pathological conditions (5). The differential phosphorylation at the two sites under high glucose exposure needs to be studied further.
